# Data on physico-chemical, performance, combustion and emission characteristics of Persea Americana Biodiesel and its blends on direct-injection, compression-ignition engines

**DOI:** 10.1016/j.dib.2018.10.166

**Published:** 2018-11-03

**Authors:** P.A.L. Anawe, Folayan J. Adewale

**Affiliations:** a Department of Petroleum Engineering, College of Engineering, Covenant University, Ota, Nigeria; b Department of Petroleum Engineering, University of Ibadan, Nigeria

**Keywords:** Biodiesel, Combustion characteristics, Emission characteristics, Performance characteristics, Persea americana, Smoke opacity, Transesterification reaction

## Abstract

This data article is based on research work which examines the potential of Persea americana (Avocado) plant oil biodiesel and its blends as viable alternative to the environmentally degrading and virtually unsustainable fossil fuel (diesel) in direct-injection, compression-ignition engines. The Avocado biodiesel was synthesized by a single-process, methanol-catalyzed transesterification reaction under optimum conditions. The cold flow and critical properties of the produced biodiesel and its blends were analyzed by using America standard for testing materials (ASTM) procedures.

Data on performance and combustion characteristics of the biodiesel and its blends on test engine HR178FA/FAE Single Cylinder, 4-Stroke, air-cooled, direct-injection, compression-ignition diesel engine at various engine loads of 0%, 20%, 40%, 60%, 80% and 100% are provided. The emission and smoke opacity characteristics were measured by a nova 7460 exhaust gas analyzer and AVL 437 smoke meter respectively.

**Specifications table**

**Table:** 

Subject area	Chemical Engineering
More specific subject area	Bio engineering
Type of data	Tables, figures, images, graphs
How data was acquired	The performance and combustion characteristics of the biodiesel and its blends on diesel engines were determined by using **HR178FA/FAE Single Cylinder,4-Stroke, air-cooled, direct-injection diesel engine.** While the emission and smoke opacity characteristics were measured by A nova 7460 exhaust gas analyzer and AVL 437 smoke meter respectively.
Data format	Raw, analyzed
Experimental factors	Fuel performance characteristics depends on cetane number, cold flow properties (pour and cloud points), heating value, density, flash point, kinematic viscosity, iodine value, acid value and amount of sulphated ash.
Experimental features	The Avocado biodiesel was produced in the laboratory by a process known as transesterification. The cold flow and critical properties of the produced biodiesel were evaluated by using the American Society for Testing Materials (ASTM) and European committee for standardization (EN).
Data source location	Nigeria.
Data accessibility	Data are available within this article
Related research article	None

**Value of the data**

• The data showed the technical viability and environmental friendliness potential of Avocado oil as new discovery and promising candidate in the endless search for biodiesel fuel by the community of scientists and engineers.
• The data can be used to examine the effect of blending on the improvement of biodiesel critical properties as well as performance, combustion and emission characteristics.
• The data are of immense importance to the scientific community because it analyzed and compared the quality of biodiesel produced from Avocado oil with that of conventional diesel by using American Society for Testing Materials (ASTM) and the European (EN) standard procedures and techniques.
• The data can be used by the scientific community to compare the amount of oxides of nitrogen (NOX), carbon monoxide (CO) and unburnt hydrocarbons (HCs) that are being produced by the burning of the avocado biodiesel and its blends with that of diesel.
• The data provide information for the larger scientific community regarding the amount of soot content in the produced biodiesel fuel exhaust and its blends and compares it with that of diesel fuel exhaust.

## 1 Data

The data obtained from this research work comes from the experimental investigation of physico-chemical, performance, combustion and emission characteristics of Persea Americana biodiesel and its blends on direct-injection, compression-ignition engines.

Table 1 showed the cold flow properties (cloud point and pour point) results of the produced biodiesel while Table 2 represents data on the critical properties of all the fuel samples. The critical properties are kinematic viscosity at 40 °C, specific gravity at 15 °C, flash point, cetane number, iodine value and heating value.

**Table 1:** Cold flow properties result.

**Fuel**	**Cloud point (**°**C)**	**Pour point (**°**C)**
**D100**	**1.7**	**−2.5**
**ABD20**	**2.2**	**−3.1**
**ABD40**	**2.8**	**−3.9**
**ABD50**	**3.05**	**−4.2**
**ABD60**	**3.3**	**−4.7**
**ABD80**	**3.9**	**−5.3**
**ABD100**	**4.4**	**−5.8**
**AOD100**	**9**	**2**

**Table 2:** Critical properties result.

**Fuel**	**Specific Gravity @ 15** °**C**	**Viscosity @ 40** ^**O**^**C (mm**^**2**^**/s)**	**Flash point (**°**C)**	**Cetane number**	**Iodine value (gI**_**2**_**/100 g of fuel)**	**Heating value (kj/kg)**
**D100**	**0.834**	**2.95**	**58**	**49.3**	**38**	**45,310**
**ABD20**	**0.842**	**3.07**	**66**	**50.15**	**43**	**44,470**
**ABD40**	**O.851**	**3.22**	**78**	**51.28**	**50**	**43,364**
**ABD50**	**0.856**	**3.30**	**93**	**51.80**	**54**	**42,814**
**ABD60**	**0.862**	**3.43**	**101**	**52.6**	**59**	**42,156**
**ABD80**	**0.869**	**3.59**	**123**	**53.86**	**66**	**41,090**
**ABD100**	**0.875**	**3.75**	**148**	**55.10**	**78**	**40,106**
**AOD100**	**0.918**	**34**	**186**	**49.68**	**96**	**39,485**

The **brake specific fuel consumption (kg/kwhr)** at different engine loads is presented in Table 3 while Table 4 described the brake specific energy consumption (kj/kghr) at different engine loads.

**Table 3:** Brake specific fuel consumption (Kg/kwhr) at different engine loads.

**%Load**	**Fuel**						
	**D100**	**ABD20**	**ABD40**	**ABD50**	**ABD60**	**ABD80**	**ABD100**
**0**	**—**	**—**	**—**	**—**	**—**	**—**	**—**
**20**	**0.45**	**0.48**	**0.51**	**0.53**	**0.55**	**0.575**	**0.65**
**40**	**0.36**	**0.37**	**0.40**	**0.405**	**0.42**	**0.44**	**0.465**
**60**	**0.28**	**0.30**	**0.32**	**0.335**	**0.345**	**0.367**	**0.38**
**80**	**0.25**	**0.27**	**0.285**	**0.292**	**0.3**	**0.318**	**0.336**
**100**	**0.33**	**0.35**	**0.37**	**0.38**	**0.39**	**0.42**	**0.44**

**Table 4:** Brake specific energy consumption (kj/kghr) at different engine loads.

**%Load**	**Fuel**						
	**D100**	**ABD20**	**ABD40**	**ABD50**	**ABD60**	**ABD80**	**ABD100**
**0**	**—**	**—**	**—**	**—**	**—**	**—**	**—**
**20**	**20.5**	**21.30**	**22.15**	**22.43**	**22.85**	**23.90**	**24.75**
**40**	**14.3**	**14.85**	**15.40**	**15.65**	**15.90**	**16.40**	**16.90**
**60**	**13.15**	**13.58**	**14.10**	**14.35**	**14.60**	**15.10**	**15.50**
**80**	**12.00**	**12.45**	**12.95**	**13.20**	**13.50**	**13.98**	**14.60**
**100**	**11.30**	**11.65**	**12.05**	**12.25**	**12.40**	**12.80**	**13.10**

Similarly, the brake thermal efficiency in percentage at different engine loads for all the fuel samples is shown in Table 5. While Table 6 is the data on the ignition delay period in degrees at different engine loads. The exhaust gas temperature behaviour of all the fuel samples is graphically presented in Fig. 2 while oxides of nitrogen (nox) emission characteristics at different engine loads is shown in Fig. 3. The emission characteristics of carbon II oxide and unburnt hydrocarbons at different engine loads of 0%, 20%, 40%, 60%, 80% and 100% are presented in Figs. 4 and 5 respectively.

**Table 5:** Brake thermal efficiency (%) at different engine loads.

**%Load**	**Fuel**						
	**D100**	**ABD20**	**ABD40**	**ABD50**	**ABD60**	**ABD80**	**ABD100**
**0**	**—**	**—**	**—**	**—**	**—**	**—**	**—**
**20**	**15.40**	**14.80**	**14.25**	**13.94**	**13.55**	**13.06**	**12.50**
**40**	**23.50**	**22.75**	**21.96**	**21.55**	**21.10**	**20.30**	**19.47**
**60**	**27.60**	**26.98**	**26.60**	**26.30**	**26.04**	**25.27**	**24.75**
**80**	**31.00**	**30.40**	**29.70**	**29.37**	**28.95**	**28.35**	**27.60**
**100**	**35.20**	**34.35**	**33.50**	**33.08**	**32.65**	**31.90**	**31.20**

**Table 6:** Ignition delay period (degrees) at different engine loads.

**%Load**	**Fuel**						
	**D100**	**ABD20**	**ABD40**	**ABD50**	**ABD60**	**ABD80**	**ABD100**
**0**	**24.50**	**24.30**	**24.05**	**23.90**	**23.75**	**23.40**	**22.86**
**20**	**22.70**	**22.40**	**22.10**	**21.95**	**21.60**	**21.50**	**21.15**
**40**	**20.80**	**19.65**	**19.25**	**19.05**	**18.80**	**18.60**	**17.90**
**60**	**18.15**	**17.80**	**17.50**	**17.30**	**17.15**	**16.80**	**16.45**
**80**	**17.20**	**16.75**	**16.30**	**15.75**	**15.85**	**15.46**	**15.18**
**100**	**16**	**15.60**	**15.20**	**14.95**	**14.80**	**14.38**	**13.94**

**Fig. 1:**
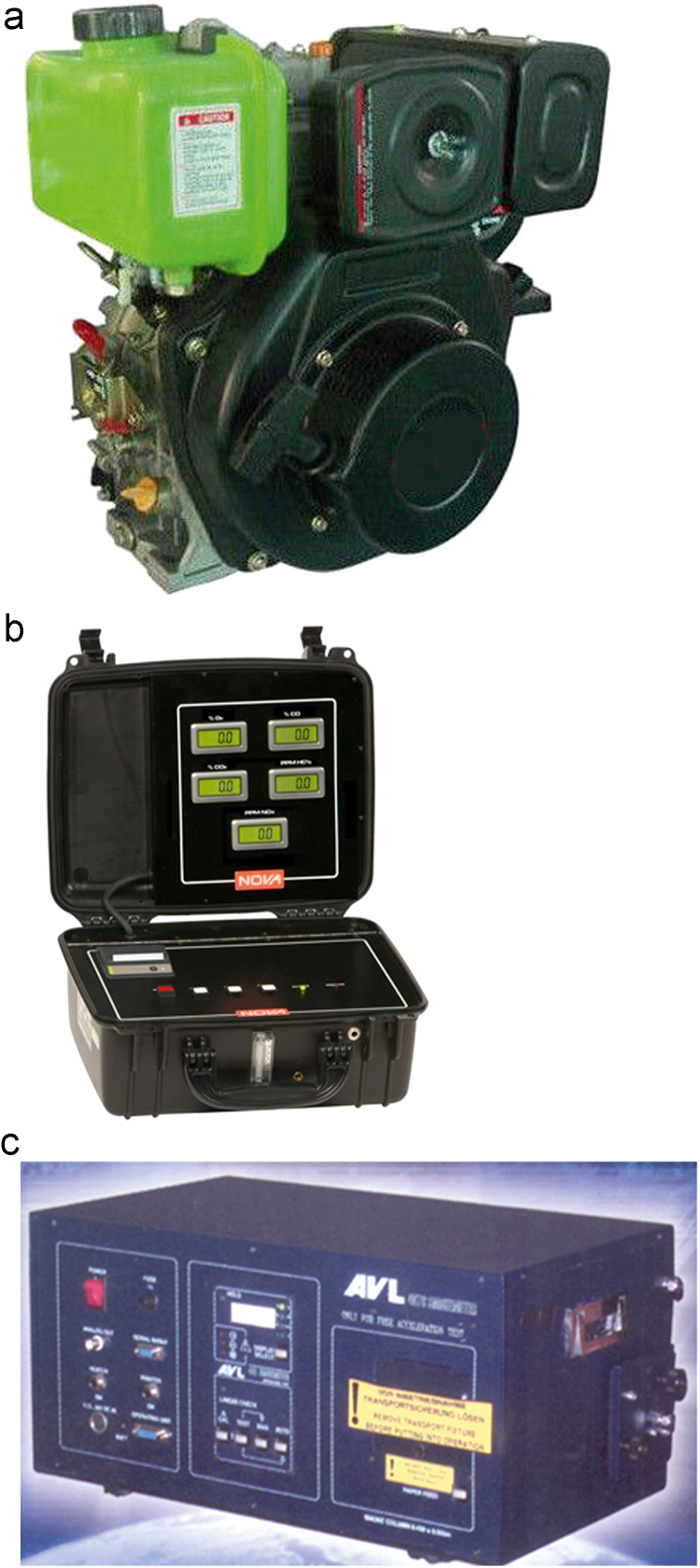
a: HR178FA/FAE Single cylinder, 4-Stroke, air-cooled, direct-injection diesel engine. b: NOVA 7460 series portable engine exhaust gas analyzer c: AVL 437 Smoke meter.

**Fig. 2:**
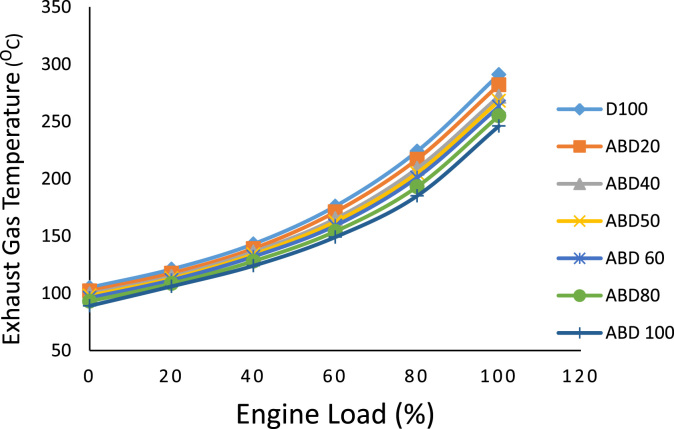
Exhaust gas temperature behaviour at different engine loads.

**Fig. 3:**
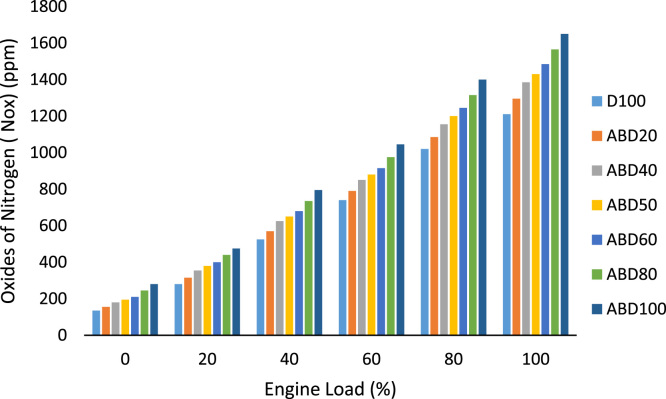
Oxides of nitrogen (Nox) emission characteristics at different engine loads.

**Fig. 4:**
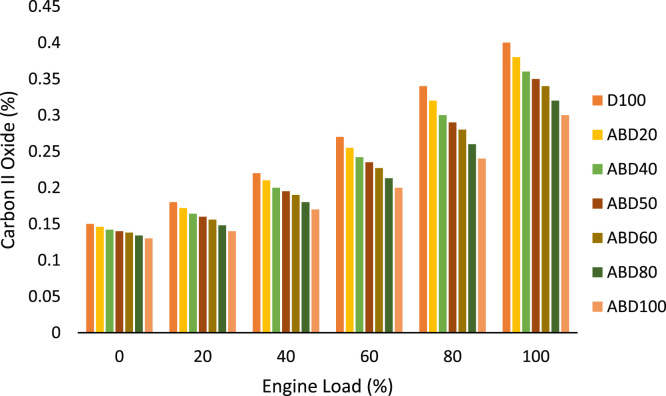
Carbon II oxide emission characteristics at different engine loads.

**Fig. 5:**
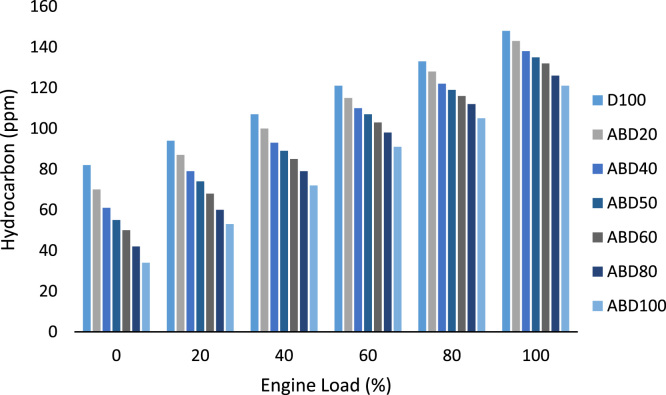
Hydrocarbon emission characteristics at different engine loads.

**Fig. 6:**
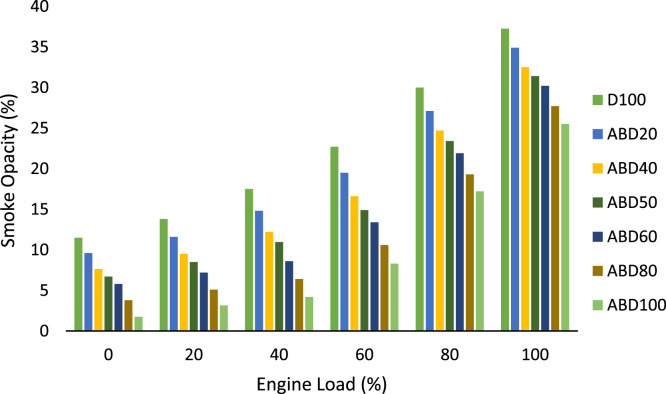
Smoke opacity characteristics at different engine loads.

Finally, Fig. 6 is the smoke opacity characteristics of the biodiesel and its blends at different engine loads.

## 2 Experimental design, materials, and methods

The Avocado biodiesel was produced in the laboratory by a process known as transesterification [1], [2], [3], [4], [5], [6], [7], [8], [9]. The process involved a catalyst concentration of 1.00% sodium hydroxide (NaOH), operating temperature of 65 °C, methanol to oil molar ratio of 6:1 and a reaction time of 2 h for optimum biodiesel yield production [10], [11], [12]. The cold flow and critical properties of the produced biodiesel were evaluated by using the American Society for Testing Materials (ASTM) and European committee for standardization (EN) [13], [14].

The produced biodiesel was then blended with varying proportion of diesel and thoroughly mixed with a multimixer according to the following nomenclature: **D100** (100% Diesel + 0% Biodiesel), **AOD100** (100% Avocado Oil), **ABD100** (100% Avocado Biodiesel + 0% Diesel), **ABD80** (80% Avocado Biodiesel + 20% Diesel), **ABD60** (60% Avocado Biodiesel + 40% Diesel), **ABD50** (50% Avocado Biodiesel + 50% Diesel), **ABD40** (40% Avocado Biodiesel + 60% Diesel), **ABD20** (20% Avocado Biodiesel + 80% Diesel).

The test engine is a single cylinder, 4-stroke, air cooled direct injection-compression ignition engine **(**Fig. 1**a)** with 4.5 KW power rating, compression ratio of 20:1, bore-stroke of 78 mm × 64 mm and speed of 3,600 rpm. Attached to the engine are dynamometer with a controller and torque-meter for loading and torque measurement respectively. A nova 7460 exhaust gas analyzer with measurement range of 0–10% CO, 0–2000 ppm NOX and 0–2000 ppm HCs shown in Fig. 1**b** was used to measure NOx, CO and H/Cs emissions from the test engine. Similarly, a filter type AVL 437 smoke meter with measurement range of 0–100% and ±1 full scale reading accuracy and reproducibility shown in Fig. 1**c** was used for the measurement of the soot content in each fuel exhaust. The smoke meter has a twenty minutes heating time with maximum smoke temperature of 210 °C.

The exhaust gas temperature was measured by a thermocouple which was placed in the exhaust manifold. Combustion analyses software in a computer was used to measure the ignition delay period.

## References

[1] knothe G., Dunn R.O., Bagby M.O. (1997). Biodiesel: the use of vegetable oils and their derivatives as alternative diesel fuels. Fuels and Chemical Biomass.

[2] Ayoola A.A., Efeovbokhan V.E., Adeeyo A.O., Johnson O.O. (2013). Methanolysis of Triglyceride using jatropha oil and KOH catalyst. Int. J. Adv. Res. IT Eng..

[3] Efeovbokhan V.E., Ayoola A.A., Anawe P.A.L., Ogheneofego O. (2012). The effects of trans-esterification of castor seed oil using ethanol, methanol and their blends on the properties and yields of biodiesel. Int. J. Eng. Technol..

[4] Owolabi R.U., Osiyemi N.A., Amosa M.K., Ojewumi M.E. (2011). Biodiesel from house hold/restaurant waste cooking oil. J. Chem. Eng. Process Technol..

[5] Agarwal A.K. (1998). Vegetable oils versus diesel fuel: development and use of biodiesel in a compression ignition. TERI Inf. Dig. Energy. (TIDE).

[6] Van-gerpen J., Shanks B., Pruszko R., Clements D., Knothe G. (2004). Biodiesel Analytical Methods.

[7] Van-gerpen J., Shanks B., Pruszko R., Clements D., Knothe G. (2004). Biodiesel Production Technology.

[8] Woolf A., Wong M., Eyres L., McGhie T., Lund C., Olsson S., Wang Y., Bulley C., Wang M., Friel E., Raquejo-Jackman C., Moreau R., Kamal-Eldin A. (2009). Avocado oil from cosmetic to culinary oil. Gourment and Health promoting Specialty Oils.

[9] Uriate F.A. (2010). Biofuels from plant oils: a book for practitioners and professionals involved in biofuels, to promote a better and more accurate understanding of the nature, production and use of biofuels from plant oils.

[10] Encinar J.M., Gonzalez J.F., Rodriguez-Reinares A. (2007). Ethanolysis of used frying oil biodiesel preparation and characterization. Fuel Process. Technol..

[11] Musa I.A. (2016). The effect of alcohol to oil molar ratios and the type of alcohol on biodiesel production using transesterification process. Egypt. J. Pet..

[12] Al Naggar M.M., Ashour F.H., Ettouney R.S., El Rifai M.A. (2017). Production of biodiesel from locally available spent vegetable oil. J. Renew. energy Sustain. Dev..

[13] American Society for Testing Materials (2007). ASTM standard specification for biodiesel fuel (B100): annual book of ASTM Standards.

[14] European committee for standardization (2003). Committee for standardization of automotive fuels-fatty acid FAME for diesel engines requirements and test methods.

