# Supplementary material for: Data on physico-chemical, performance, combustion and emission characteristics of Persea Americana Biodiesel and its blends on direct-injection, compression-ignition engines
Source: Data Brief. 2018 Nov 3;21:1533–40. doi: 10.1016/j.dib.2018.10.166 (PMC6240667; doi:10.1016/j.dib.2018.10.166)
Supplement: Supplementary file 2 — Supplementary material [file mmc2.pdf]

COVENANT UNIVERSITY
CENTRE FOR LEARNING RESOURCES
CERTIFICATE OF SIMILARITY CHECK

This is to certify that the article titled **“Data On Physico-chemical, Performance, Combustion and Emission Characteristics of Persea Americana Biodiesel and its Blends on Direct-Injection, Compression-Ignition Engines.”** submitted by **Anawe A. L Paul ; Folayan J. Adewale** has been checked to ascertain the level of plagiarism using TURNITIN. The Similarity Index of **12%** is adjudged okay for further processing.

Thank You.

COVENANT UNIVERSITY
CENTRE FOR LEARNING
RESOURCES
DIRECTOR
DATE

ILO, P. I. (PhD)

DCLR
